# Dendritic Mesoporous Silica Nanoparticle Supported PtSn Catalysts for Propane Dehydrogenation

**DOI:** 10.3390/ijms232112724

**Published:** 2022-10-22

**Authors:** Ning Zhang, Yiou Shan, Jiaxin Song, Xiaoqiang Fan, Lian Kong, Xia Xiao, Zean Xie, Zhen Zhao

**Affiliations:** 1 Institute of Catalysis for Energy and Environment, Shenyang Normal University, Shenyang 110034, China; 2 State Key Laboratory of Heavy Oil Processing, China University of Petroleum, Beijing 102249, China

**Keywords:** propane dehydrogenation, dendrimer-like silica nanoparticle support, Pt/Sn ratios

## Abstract

PtSn catalysts were synthesized by incipient-wetness impregnation using a dendritic mesoporous silica nanoparticle support. The catalysts were characterized by XRD, N_2_ adsorption–desorption, TEM, XPS and Raman, and their catalytic performance for propane dehydrogenation was tested. The influences of Pt/Sn ratios were investigated. Changing the Pt/Sn ratios influences the interaction between Pt and Sn. The catalyst with a Pt/Sn ratio of 1:2 possesses the highest interaction between Pt and Sn. The best catalytic performance was obtained for the Pt_1_Sn_2_/DMSN catalyst with an initial propane conversion of 34.9%. The good catalytic performance of this catalyst is ascribed to the small nanoparticle size of PtSn and the favorable chemical state and dispersion degree of Pt and Sn species.

## 1. Introduction

Propene is an important raw material that is widely used in the production of polypropene, propene oxide, acrylonitrile, etc. Currently, it is mainly obtained by steam cracking of naphtha, and the fluid catalytic cracking process. Due to the restriction of the oil price and storage and the growing demand for propene, some alternative methods, including the methanol-to-olefins (MTO) reaction and the dehydrogenation of propane (PDH) process, have been used to increase its production [1]. Among them, PDH is advantageous because of the price gap between propene and propane and the abundant resources of shale gas and natural gas. The PDH process is endothermic. In order to obtain a desirable yield of propene, the PDH process operates at a high temperature, above 450 °C. The high temperature is favorable for coke formation and hydrocarbon cracking, which increase the deactivation of catalysts. Therefore, the development of PDH catalysts with high catalytic activity and stability is key. Two main types of catalysts are used for PDH: noble metal-based and non-noble metal-based catalysts. Cr-based catalysts, as the most prominent example of non-noble metal-based catalysts with high catalytic activity for PDH, have been commercialized. Other non-noble metal-based catalysts including Zn [2], Mo [3], V [1,4], Co [5], Ga [6,7], Fe [8,9], Cu [10], etc. [11,12,13] also show catalytic activity for PDH. As the extensively investigated precious metal catalyst for PDH, Pt-based catalysts are also used in commercial processes. However, Pt-based catalysts still face the challenge of deactivation due to coke deposition and Pt nanoparticle sintering. In order to improve the stability of Pt-based catalysts, many strategies have been adopted, including adding metal promoters. Sn is the most effective promoter for the Pt catalyst in PDH [14,15,16,17,18]. The role of Sn is mainly explained in terms of geometric effects and electronic effects [19]. It can assist in the separation of large Pt ensembles into small clusters, which can suppress structure-sensitive side reaction. The electron transfer between Sn and Pt atoms can change the Pt electron density, thereby affecting the adsorption–desorption of reactants and products, promoting the selectivity and stability. Sexton et al. [20] found that Sn(II) is a surface modifier of γ-Al_2_O_3_ and the change of electronic interactions between Pt and Sn(II)-γ-Al_2_O_3_ changes the reactivity. Motagamwala et al. [21] reported a silica-supported Pt_1_Sn_1_ nanoparticle catalyst with excellent stability during PDH. Xiong et al. [22] found that the formation of small PtSn clusters allows the catalyst to achieve high propene selectivity due to the facile desorption of propene. Wang et al. [23] described the structural evolution of Pt–Sn bimetallic nanoparticles for PDH and found the recovery of Pt–Sn alloy by Sn segregation important for sustaining the high catalytic performance of PDH. Liu et al. [24] also investigated the evolution of Pt and Sn species during high-temperature treatments. Gao et al. [25] reported that Pt clusters that were partially modified by reduced Sn exhibited higher activity than PtSn alloy counterparts for PDH. Although Sn as the promoter has been widely studied, the nature of the PtSn active sites still needs further research. Moreover, Zn [26,27], Cu [28], Fe [29,30], Co [31], Ga [32], In [33,34] and Mn [35,36] are also used as promoters in PDH. The other strategy is to improve metal–support interactions so as to increase the catalytic performance. Many supports have been used, including Al_2_O_3_, SiO_2_ and zeolites [37,38,39,40,41]. Compared with Al_2_O_3_ and zeolites, SiO_2_ shows chemical inertness and is suitable for use as a support to study the relationship between active components and catalytic performance.

Dendritic mesoporous silica nanoparticles (DMSNs) are attractive materials as support due to their unique feature. They show high surface area, which mainly originates from their dendrimer-like morphology, not from their mesoporous channels as with SBA-15 or MCM-41 [42]. This unique feature shows the usefulness compared to conventional ordered mesoporous silica for some catalytic reactions [43]. Moreover, the monodispersed nanoparticles with a short diffusion length as the support might promote the diffusion of reactants and products and influence the catalytic behavior. It has been reported that support with suitable physical morphology is of fundamental importance for PDH. Compared with SBA-15 or MCM-41, the diffusion distance in the pores is shortened on DMSN support (radius of the nanoparticle about 100 nm for DMSN and usually several μm for SBA-15 or MCM-41) [44]. This makes the PtSn nanoparticles in the pores easily accessible for reactants and makes the products easy to diffuse to avoid further reaction. Compared with the zeolites of micropores, the PtSn nanoparticles are usually loaded on the external surface area rather than in the microporous area of zeolites, which will decrease the confined effect of the pores. Therefore, in this paper, DMSNs are prepared as the support and used in a PDH reaction. Catalysts with different ratios of Pt and Sn were prepared by the impregnation method and were characterized by means of various techniques to investigate the dispersion and state of Pt and Sn species on different ratios of Pt and Sn. The catalytic performances were tested with PDH to investigate the influence of a PtSn active component on the catalytic performance.

## 2. Results and Discussion

### 2.1. Analysis of Physico-Chemical Properties

**Figure 1:**
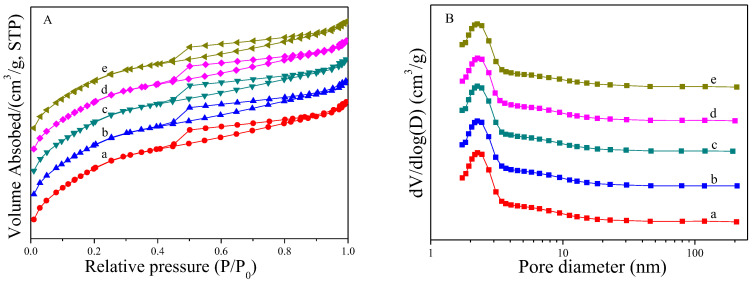
(**A**) N_2_ adsorption–desorption isotherms and (**B**) pore size distributions of the calcined PtSn/DMSN catalysts with different ratios of Pt and Sn: (a) Pt/DMSN, (b) Pt_1_Sn_1_/DMSN, (c) Pt_1_Sn_2_/DMSN, (d) Pt_1_Sn_3_/DMSN, (e) Pt_1_Sn_4_/DMSN.

Nitrogen sorption measurements were conducted to characterize the pore parameters of the catalysts with different ratios of Pt and Sn. The corresponding nitrogen adsorption–desorption isotherms and pore size distributions are shown in Figure 1A,B, respectively. All the catalysts showed typical type IV adsorption–desorption isotherms with H_4_-like hysteresis loops at values of P/P_0_ ranging from 0.40 to 1.00, which indicated the presence of mesopores. The similarity of the catalysts demonstrated that the loading of metals did not cause damage to the porous structure of the support. Figure 1B shows the pore size calculated according to the BJH method. The pore sizes of these samples through Pt/DMSN to Pt_1_Sn_4_/DMSN catalyst were found to be consistent with the value of 3.1 or 3.2 nm. This may be caused by the low metal loading amounts or the high dispersion of the metals.

According to the adsorption–desorption isotherms, the textural properties of these samples were calculated by specific algorithms, and the results are shown in Table 1. The Pt/DMSN sample showed a surface area and total pore volume of 1091.8 m^2^/g and 0.79 cm^3^/g, respectively. The high surface area could be attributed to the fine features in dendritic spheres in the form of wrinkles or lamellar structures [45], and the pore volume was due to the plentiful pore construction in the DMSN support. With the increasing of Sn loadings, the BET surface area of the catalysts decreased from 1091.8 m^2^/g on the Pt/DMSN catalyst to 1005.5 m^2^/g on the Pt_1_Sn_4_/DMSN catalyst. The decrease in surface area with the increasing of Sn loadings indicated that the metals entered into the mesopores, which hindered the nitrogen molecule diffusion into the pore, leading to a decrease in the BET surface area. It also was demonstrated by the decrease in total pore volume and mesoporous volume, which decreased from 0.79 cm^3^/g to 0.71 cm^3^/g and 0.55 cm^3^/g to 0.49 cm^3^/g, respectively. The gradual reduction in the surface area, total pore volume and mesoporous volume from the Pt/DMSN catalyst to the Pt_1_Sn_4_/DMSN catalyst may have been due to the increasing of the total loading amounts of the metals. Moreover, the pore sizes of these catalysts were nearly unchanged, which implies the formation of nanoparticles in the mesopores.

**Figure 2:**
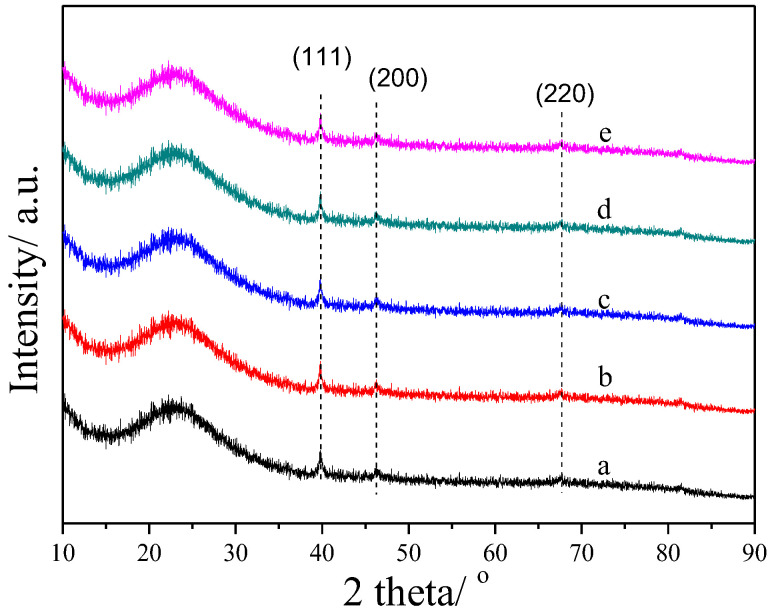
XRD patterns of calcined PtSn/DMSN catalysts with different ratios of Pt and Sn: (a) Pt/DMSN, (b) Pt_1_Sn_1_/DMSN, (c) Pt_1_Sn_2_/DMSN, (d) Pt_1_Sn_3_/DMSN, (e) Pt_1_Sn_4_/DMSN.

To investigate the influence of the ratios of Pt and Sn on the crystalline structure of Pt and Sn, XRD characterization was carried out; the results are shown in Figure 2. It can be seen that the catalysts with different ratios of Pt and Sn showed similar diffraction peaks in the range of 10° to 90°, and the intensity of these peaks was considerable. The broad diffraction peak at around 23.5° was a typical reflection of amorphous silica. The weak peaks at 2θ of 39.8°, 46.4°, 67.7° and 81.4° were assigned to the (111), (200), (220) and (311) reflections of cubic Pt, respectively [46]. The similar intensity of these peaks indicated the similar crystallinity of Pt, which indicated that the ratio of Pt and Sn (the amounts of Sn) had a negligible influence on the size of Pt. Moreover, there were no apparent diffraction peaks attributed to SnO_x_ in the XRD patterns, indicating high dispersion of SnO_x_ or the formation of amorphous SnO_x_ phases.

**Figure 3:**
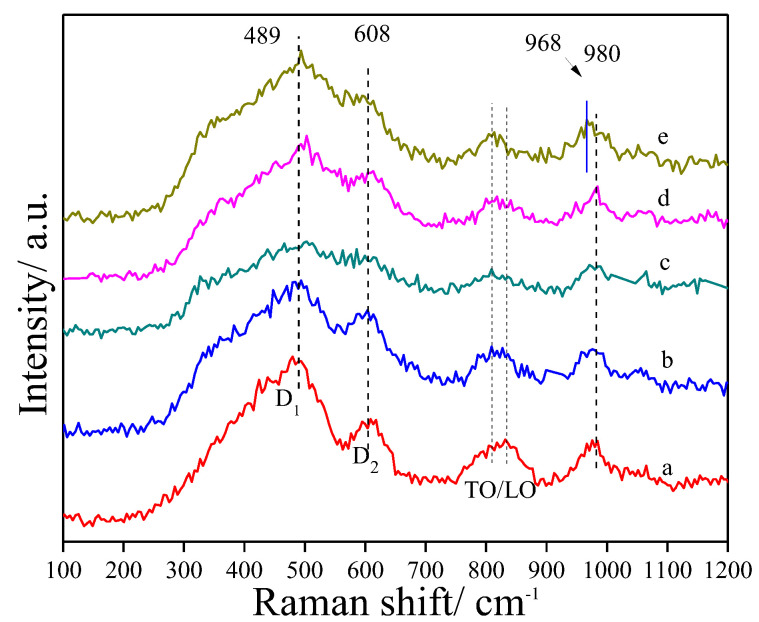
Raman spectra of calcined PtSn/DMSN catalysts with different ratios of Pt and Sn: (a) Pt/DMSN, (b) Pt_1_Sn_1_/DMSN, (c) Pt_1_Sn_2_/DMSN, (d) Pt_1_Sn_3_/DMSN, (e) Pt_1_Sn_4_/DMSN.

Raman spectra of fresh catalysts with different Pt and Sn ratios are presented in Figure 3. It can be seen that the spectra of PtSn/DMSN samples showed typical vibrations of SiO_2_ in the range of 100–1200 cm^−1^ [47]. For the Pt/DMSN catalyst, there was no diffraction peaks attributed to Pt species, indicating the Pt species existing in the form of the metallic state. For the catalysts with high ratios of Pt and Sn (Pt/Sn < 1/4), the Raman spectra for the PtSn/DMSN catalysts showed no obvious changes, which may have been caused by the low metal loading amounts. However, for Pt_1_Sn_4_/DMSN, a slight redshift of about 12 cm^−1^ was noticed for the band at 980 cm^−1^, which suggests the incorporation of Sn in the framework of silica. This redshift could be due to an appreciable decrease in the bond strength and/or bond angle caused by the formation of oxygen–metal–oxygen (O–Sn–O) bridges in the silica framework [48]. Li et al. [49] reported that the band at about 960 cm^−1^ was associated with Si–O–Si linkages next to M–O–Si bonds and identified contributions attributed to transition-metal ions bonded to the framework. It can be seen from the Raman spectra that Sn incorporated into the framework of silica resulted in a strong interaction between Sn and the support. The strong interaction between Sn and the support can favor the interaction between Pt and the support so as to increase the stability of the PtSn/DMSN catalyst during the PDH reaction.

**Figure 4:**
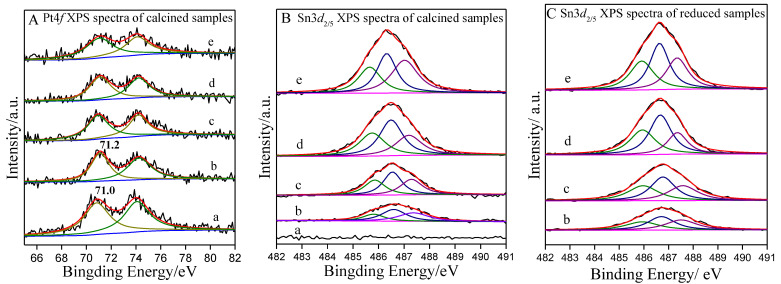
Pt4*f* (**A**) and Sn3*d*_5/2_ (**B**,**C**) XPS spectra of calcined and reduced PtSn/DMSN catalysts with different ratios of Pt and Sn: (a) Pt/DMSN, (b) Pt_1_Sn_1_/DMSN, (c) Pt_1_Sn_2_/DMSN, (d) Pt_1_Sn_3_/DMSN, (e) Pt_1_Sn_4_/DMSN.

The chemical state of Pt and Sn species on the catalysts were examined by XPS. The XPS spectra of Pt4*f* and Sn3*d*_5/2_ are presented in Figure 4. As shown in Figure 4A, the Pt4*f* spectra of all the PtSn/DMSN catalysts could be deconvoluted into two peaks that could be assigned to metallic Pt (Pt°) [50], which indicated that the Pt species existed in a metallic state on the PtSn/DMSN catalysts. This was also in agreement with the XRD results. Moreover, the binding energy for Pt/DMSN was 71.0 eV, while the binding energy for PtSn/DMSN was 71.2 eV. The slight shift to high binding energy in PtSn/DMSN catalysts indicated the electronic effects between Pt and Sn [51]. Figure 4B shows the Sn3*d*_5/2_ XPS spectra of calcined samples. There were three peaks at 485.8, 486.6 and 487.3 eV after deconvolution, corresponding to the Sn°, Sn^2+^ and Sn^4+^ species, respectively [52]. This indicated that the Sn species on the catalysts existed both in a metallic state and in the form of SnO*_x_*. Figure 4C shows the Sn3*d*_5/2_ XPS spectra of reduced samples. It can be seen that the Sn species still existed both in a metallic state and in the form of SnO*_x_* after reduction treatment. Furthermore, the fraction of metallic Sn species increased compared with the calcined samples. According to the XPS results, it can be seen that the state of Pt species on PtSn/DMSN catalysts with different ratios of Pt and Sn was a metallic state, while the state of Sn species was both an oxidation state and a metallic state.

**Figure 5:**
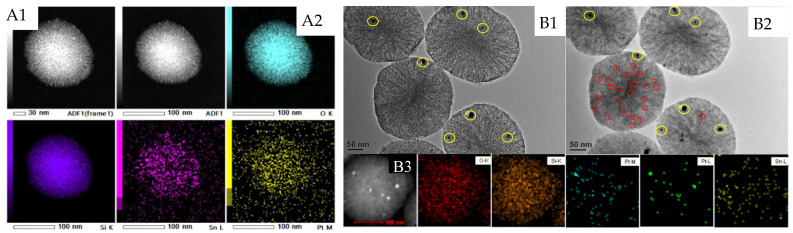
TEM images and EDS elemental mapping of calcined (**A1**,**A2**) Pt_1_Sn_2_/DMSN and (**B1**–**B3**) Pt_3_Sn_9_/DMSN catalyst.

To investigate the morphology of PtSn/DMSN and to provide evidence for the distribution of Pt and Sn, TEM analyses were carried out. Figure 5A1 shows the TEM images of the Pt_1_Sn_2_/DMSN catalyst. It can be seen that the morphology of the DMSN was porous spheres with a dendritic pore structure arranged in three dimensions to form spheres. The unique hierarchical pore structures gave such DMSN material potential as a catalyst support. There were some nanoparticles that appeared in the DMSN support, which may be assigned to PtSn nanoparticles. The EDS elemental mapping in Figure 5A2 shows that Si, O and Sn species were uniformly dispersed on the DMSN support. In order to investigate the interaction between metal and the support, the high loading PtSn/DMSN catalyst was prepared and characterized by TEM. Figure 5B1 shows the TEM images of Pt_3_Sn_9_/DMSN with the loading amounts of 3 wt% of Pt and 9 wt% of Sn. It can be seen that the sizes of the nanoparticles were larger than those of Figure 5A1 because of the increasing of Pt and Sn loading amounts. In order to investigate the dispersion of Pt and Sn, EDS elemental mapping was carried out. The EDS elemental mapping in Figure 5B3 of Pt apparently showed a hot spot, indicating the aggregation of Pt. It can be seen from Figure 5B3 that the Pt elemental mapping showed corresponding dispersion with the STEM image, while Sn elemental mapping showed more uniform dispersion with no apparent hot spot shown corresponding to the nanoparticles in the STEM images. It was indicated that the dispersions of Pt and Sn were different. Sn species possessed strong interactions with the support so as to show highly dispersion, which was demonstrated by the Raman results that some of Sn species were incorporated into the framework of the silica. Pt species, by contrast, possessed weak interactions with the support so as to show lower dispersion than Sn species. Furthermore, the strong interaction of Sn and the support may have been favorable to the interaction of Pt and the support so as to increase the dispersion of Pt. Figure 5B2 is Figure 5B1 under several minutes of high voltage exposure. It can be seen that a large amount of small nanoparticles are shown (the red circle). This indicates that there were both highly dispersed small nanoparticles and nanoparticles (yellow circle) in the Pt_3_Sn_9_/DMSN sample. Therefore, there were both Pt and Sn element dispersions on the DMSN support except the hot spot. Furthermore, the uniform dispersal of Pt and Sn in Figure 5A2 indicates the high dispersion of both Pt and Sn, which may have been caused by the strong interactions of Sn and the support.

### 2.2. Catalytic Performance of Propane Dehydrogenation

**Figure 6:**
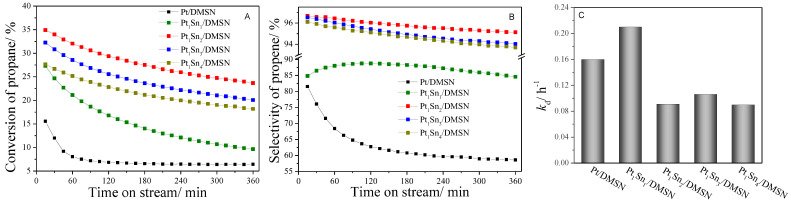
Propane conversion (**A**) and propene selectivity (**B**) during 6 h PDH over PtSn/DMSN catalysts; (**C**) the deactivation rate constant (*k*_d_) of PtSn/DMSN catalysts.

The PtSn/DMSN catalysts were subjected to the propane dehydrogenation reaction. Figure 6 shows the catalytic performances of PtSn/DMSN catalysts with different ratios of Pt and Sn. As shown in Figure 6A, the initial conversion of propane over the Pt/DMSN catalyst was 15.6%, indicating that Pt works to activate propane. Compared with Pt/DMSN, the catalysts loading both Pt and Sn showed higher initial propane conversion. The initial propane conversion was 27.3% for the Pt_1_Sn_1_/DMSN catalyst, and it increased to 34.9% for the Pt_1_Sn_2_/DMSN catalyst. It decreased to 32.2% for the Pt_1_Sn_3_/DMSN catalyst. With a further increase in the amount of Sn, the initial propane conversion decreased to 27.6% for the Pt_1_Sn_4_/DMSN catalyst. The lowest initial propane conversion and fast deactivation rate (*k*_d_ = 0.21) were obtained for the Pt_1_Sn_1_/DMSN catalyst. As shown in Figure 6A, the Pt_1_Sn_2_/DMSN catalyst showed the highest propane conversion, even after 6 h of reaction. Compared with high initial propane conversions, high stabilities are more favorable for non-oxidative dehydrogenation, and high selectivity is essential for high stability. Figure 6B shows the propene selectivity for the PtSn/DMSN catalysts. Of these PtSn/DMSN catalysts, the lowest propene selectivity was obtained for the Pt/DMSN catalyst. Compared with Pt/DMSN, all PtSn/DMSN catalysts showed high propene selectivity. This indicates that the Sn promoter could increase the propene selectivity. As shown in Figure 6B, the selectivity of propene was below 90% for the Pt_1_Sn_1_/DMSN catalyst, while the selectivity of propene was higher than 95% for the Pt_1_Sn_2_/DMSN, Pt_1_Sn_3_/DMSN and Pt_1_Sn_4_/DMSN catalysts. This indicates that the low Sn loading catalyst showed lower propene selectivity compared with the high Sn loading catalyst. Moreover, the selectivity decreased from the Pt_1_Sn_2_/DMSN catalyst to the Pt_1_Sn_4_/DMSN catalyst. One reason for this tendency may be the excess Sn results in the side reaction.

In order to analyze the effect of the Pt/Sn ratio on the PDH catalytic performance, the relationship of Pt/Sn ratios over deactivation rate constant *k*_d_ was investigated and can be seen in Figure 6C. It can also be seen that *k*_d_ was 0.21 for the Pt_1_Sn_1_/DMSN catalyst, indicating fast deactivation. When the ratio of Sn to Pt increased to 2, *k*_d_ apparently decreased. The catalyst with a Pt/Sn ratio of 1/2 showed a *k*_d_ of 0.09, indicating high stability among these catalysts. Compared with the Pt_1_Sn_1_/DMSN catalyst, the catalysts with high loading amounts of Sn showed high stability.

Table 2 lists the catalytic data of PtSn/SiO_2_ catalysts used in the PDH reaction. It can be seen that the reaction conditions were different over the PtSn/SiO_2_ catalysts. Because the propane dehydrogenation reaction is an endothermic reversible reaction, the reaction temperature and feed composition resulted in different equilibrium conversions of propane. Table 2 lists the equilibrium conversion over the different conditions. It can be seen that the initial propane conversion was lower than the equilibrium conversion, and the initial propane conversion was about 85% of the equilibrium conversion in this work, indicating high activity of the Pt_1_Sn_2_/DMSN catalyst. On the other hand, the *k*_d_ of the Pt_1_Sn_2_/DMSN catalyst seemed high because of the feed composition of pure propane.

### 2.3. Characterization Results of the Spent Catalysts

**Figure 7:**
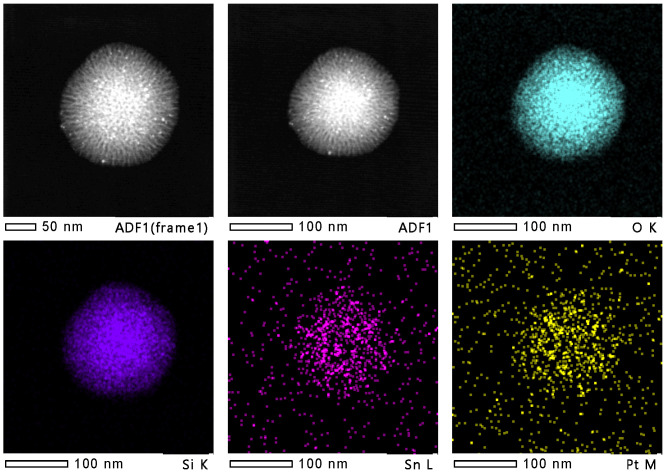
HRTEM images and EDS elemental mapping of spent Pt_1_Sn_2_/DMSN catalyst.

To provide evidence for the distribution of Pt and Sn species before and after the PDH reaction, HRTEM analyses were carried out on the spent Pt_1_Sn_2_/DMSN catalyst. Figure 7 shows the HRTEM images of the EDS elemental mapping of the spent Pt_1_Sn_2_/DMSN catalyst. Compared with Figure 5A1, the morphological structure of the spent Pt_1_Sn_2_/DMSN catalyst was similar to that of the calcined sample after a 6 h PDH reaction, indicating the high stability of the DMSN support. No significant aggregation occurred on the PtSn nanoparticles compared with the calcined sample.

**Figure 8:**
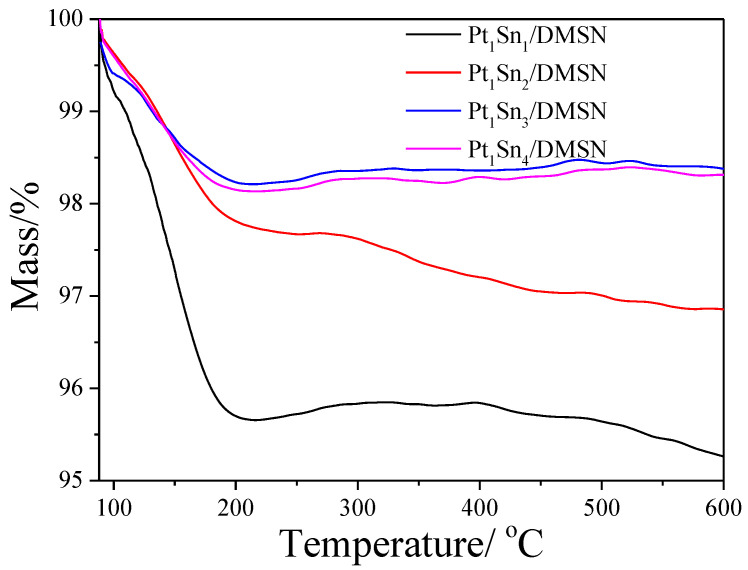
TG profiles of the spent PtSn/DMSN catalysts with different ratios of Pt and Sn.

In order to investigate the influence of coke deposition on the PDH catalytic performance, the amount of coke on the spent catalysts was investigated by TG characterization. As shown in Figure 8, the Pt_1_Sn_1_/DMSN catalyst had a mass loss of 4.7%, which was the highest among the PtSn/DMSN catalysts, indicating the largest amount of coke deposition. This is because of the occurrence of side reactions that demonstrated the low propene selectivity of this sample. The Pt_1_Sn_2_/DMSN catalyst showed higher mass loss than that of Pt_1_Sn_3_/DMSN and Pt_1_Sn_4_/DMSN catalysts. This indicates that both the dehydrogenation reaction and the coke deposition were enhanced compared to Pt_1_Sn_3_/DMSN and Pt_1_Sn_4_/DMSN catalysts. Furthermore, the Pt_1_Sn_3_/DMSN catalyst showed the lowest mass loss, indicating the lowest amount of coke deposition on the sample.

Pt-based catalysts have been widely used in the PDH process. As the most effective promoter, Sn is usually combined with Pt in PDH. The promoting effect of Sn has been understood both geometrically and electronically. Therefore, the ratio of Pt/Sn will show some influence on the geometric and electronic effects, as confirmed by the DFT calculations [55]. The catalyst with a Pt/Sn ratio of 2 showed the best catalytic performance with propane initial conversion of 34.9%, and the deactivation rate constant on the high Sn loading PtSn/DMSN catalysts was lower. The coke analysis shown in Figure 8 indicates the inhibition by Sn of coke formation.

## 3. Materials and Methods

### 3.1. Catalyst Preparation

DMSN support was prepared by the emulsion method as described previously [30]. PtSn/DMSN catalysts were prepared by the incipient-wetness impregnation method. SnCl_4_·5H_2_O and H_2_PtCl_6_·6H_2_O precursors were dissolved in deionized water to form a solution, and powder DMSN was impregnated in the solution. After that, the mixture was sonicated for 30 min and dried at 25 °C for 24 h. After being completely dried, the catalysts were calcined at 500 °C for 4 h and reduced with H_2_ at 500 °C for 4 h. The composition of the catalyst with different ratios of Pt/Sn were 1 wt% Pt and 0, 1, 2, 3 and 4 wt% Sn. For the sake of brevity, these catalysts were named as Pt/DMSN, Pt_1_Sn_1_/DMSN, Pt_1_Sn_2_/DMSN, Pt_1_Sn_3_/DMSN and Pt_1_Sn_4_/DMSN.

### 3.2. Characterization

Nitrogen adsorption/desorption isotherms at −196 °C were recorded using a Micromeritics TriStar II 3020 porosimetry analyzer. The samples were degassed at 300 °C for 8 h prior to the measurements. Wide-angle XRD patterns were obtained by a powder X-ray diffractometer (Shimadzu XRD 6000, Kyoto, Japan) using Cu Kα (λ = 0.15406 nm) radiation with a nickel filter operating at 40 kV and 40 mA in the 2θ range of 10–90 ° at a scanning rate of 4°/min. Transmission electron microscopy (TEM) images were taken on a JEOL JEM 2100 electron microscope equipped with a field emission source at an acceleration voltage of 200 kV. The TEM samples were sonicated and well suspended in ethanol. Drops of the suspension were applied, and after drying the fine particles were well dispersed on a copper grid coated with carbon. X-ray photoelectron spectra (XPS) were recorded on a Perkin-Elmer PHI-1600 ESCA spectrometer using an Mg Kα (hv = 1253.6 eV, 1 eV = 1.603 × 10^−19^ J) X-ray source. The binding energies were calibrated using the C1s peak of contaminant carbon (BE = 284.6 eV) as an internal standard. Raman spectra were performed on a Renishaw inVia Reflex Raman spectrometer with a 325 nm laser at room temperature under ambient conditions. The amount of coke deposited was determined by a NETZSCH STA 449 F5 thermogravimetric (TG) analyzer. The samples were exposed to 20% O_2_/N_2_ flowing and oxidized from ambient temperature to 600 °C at a rate of 10 °C min^−1^.

### 3.3. Catalytic Activity Test

The PDH reaction was carried out in a conventional quartz tubular micro-reactor. Then, 0.2 g of the catalyst was placed in the center of the reactor and reduced under 10% H_2_/Ar at 500 °C for 4 h. Subsequently, the pure C_3_H_8_ with a weight hourly space velocity (WHSV) of 2.4 h^−1^ was fed to the reactor, and the reaction temperature was raised to and maintained at 590 °C. The reaction products were analyzed by using an online Agilent-7890B gas chromatograph equipped with an HP-Al_2_O_3_ capillary column for the separation of CH_4_, C_2_H_4_, C_2_H_6_, C_3_H_6_ and C_3_H_8_. The conversion of propane and the selectivity for propene were defined as follows:

$$X_{C_{3}H_{8}}=\frac{{\mathrm{CH}}_{4}{+2C}_{2}H_{4}{+2C}_{2}H_{6}{+3C}_{3}H_{6}}{{\mathrm{CH}}_{4}{+2C}_{2}H_{4}{+2C}_{2}H_{6}{+3C}_{3}H_{6}{+3C}_{3}H_{8}}\times 100\%$$

$$S_{C_{3}H_{6}}=\frac{{3C}_{3}H_{6}}{{\mathrm{CH}}_{4}{+2C}_{2}H_{4}{+2C}_{2}H_{6}{+3C}_{3}H_{6}}\times 100\%$$

The deactivation rate constant *k_d_* was calculated as follows:

$$k_{d}=\frac{\ln\left[\frac{1-X_{final}}{X_{final}}\right]-\ln\left[\frac{1-X_{initial}}{X_{initial}}\right]}{t}$$

where *X_initial_* and *X_final_* represent the propane conversion at the initial and final stages of an experiment, respectively, and *t* (h) represents the duration of the PDH reaction.

## 4. Conclusions

PtSn/DMSN catalysts were synthesized by using DMSN support. The influence of the Pt/Sn ratios was investigated. The addition of Sn could enhance the catalytic performance of PDH, and the Pt/Sn ratios affected coke deposition. The addition of Sn modified the dispersion and electronic properties of Pt. The Pt_1_Sn_2_/DMSN catalyst with a Pt/Sn ratio of 1:2 showed the highest catalytic performance with initial propane conversion of 34.9%. The good catalytic performance of the Pt_1_Sn_2_/DMSN catalyst was ascribed to the small nanoparticle size of PtSn and the favorable chemical state and dispersion degree of Pt and Sn species.

## Figures and Tables

**Table 1:** Textural properties of the calcined PtSn/DMSN catalysts with different ratios of Pt/Sn.

Samples	S _BET_^a^ (m^2^·g^−1^)	V _t_^b^ (cm^3^·g^−1^)	V _mes_^c^ (cm^3^·g^−1^)	d _BJH_^d^ (nm)
Pt/DMSN	1091.8	0.79	0.55	3.2
Pt_1_Sn_1_/DMSN	1061.9	0.75	0.53	3.1
Pt_1_Sn_2_/DMSN	1042.1	0.74	0.52	3.2
Pt_1_Sn_3_/DMSN	1018.6	0.72	0.51	3.1
Pt_1_Sn_4_/DMSN	1005.5	0.71	0.49	3.2

^a^ Calculated by the BET method. ^b^ The total pore volume was obtained at a relative pressure of 0.98. ^c^ The mesoporous volume was calculated using the BJH method. ^d^ Mesopore diameter was calculated using the BJH method.

**Table 2:** Catalytic data of PtSn/SiO_2_ catalysts used in the PDH reaction.

Catalyst	Reaction Temperature (°C)	WHSV (h^−1^)	Feed Composition	Equilibrium Conversion (%)	Initial Conversion (%)	Initial Selectivity (%)	*k*_d_ (h^−1^)	Ref
Pt/Sn-MFI	600	3.2	Pure C_3_H_8_	49.3	42	95	0.012	[17]
Pt_1_Sn_1_/SiO_2_	580	4.7	C_3_H_8_:He = 4:21	67	66.5	~99	0.008	[21]
Pt-Sn/SBA-15	600	16.5	C_3_H_8_:H_2_:N_2_ = 14:14:72	60.8	~40	~92	~0.09	[23]
K-PtSn@MFI	650	29.5	C_3_H_8_:N_2_ = 5:16	83.5	~55	~99	~0.006	[24]
4SnPt/SiO_2_	500	17.7	C_3_H_8_:H_2_:N_2_ = 2:3:95	45.7	~11	99	0.004	[53]
Pt-Sn/xAlSBA-15	600	11.8	C_3_H_8_:N_2_ = 3:10	52.7	25.5	~97	0.22	[54]
Pt_1_Sn_2_/DMSN	590	2.4	Pure C_3_H_8_	40.8	34.9	96.7	0.09	This work

## Data Availability

Data are available upon reasonable request from the corresponding authors.
